# Why Australia needs to define obesity as a chronic condition

**DOI:** 10.1186/s12889-017-4434-1

**Published:** 2017-05-23

**Authors:** C. A. Opie, H. M. Haines, K. E. Ervin, K. Glenister, D. Pierce

**Affiliations:** Department of Rural Health Graham Street Shepparton Victoria, The University of Melbourne, Victoria, 3630 Australia

**Keywords:** Obesity, Chronic condition, Australia, Care plan, Treatment management

## Abstract

**Background:**

In Australia people with a diagnosed chronic condition can be managed on unique funded care plans that allow the recruitment of a multidisciplinary team to assist in setting treatment goals and adequate follow up. In contrast to the World Health Organisation, the North American and European Medical Associations, the Australian Medical Association does not recognise obesity as a chronic condition, therefore excluding a diagnosis of obesity from qualifying for a structured and funded treatment plan.

**Body:**

The Australian guidelines for management of Obesity in adults in Primary Care are structured around a five step process -the ‘5As’: Ask & Assess, Advise, Assist and Arrange’. This article aims to identify the key challenges and successes associated with the ‘5As’ approach, to better understand the reasons for the gap between the high Australian prevalence of overweight and obesity and an actual diagnosis and treatment plan for managing obesity. It argues that until the Australian health system follows the international lead and defines obesity as a chronic condition, the capacity for Australian doctors to diagnose and initiate structured treatment plans will remain limited and ineffective.

**Conclusion:**

Australian General Practitioners are limited in their ability manage obesity, as the current treatment guidelines only recognise obesity as a risk factor rather than a chronic condition.

## Background

Recent Australian research found that 30% of a rural and regional cohort of Victorians was categorized as obese (Body Mass Index ≥30 kg/m^2^), based on self-reported height and weight [1]. A further 38% were categorized as overweight (BMI between 25 and 30 kg/m^2^) [1]. However, within the same cohort only 16% reported being advised of an obesity diagnosis [1], illustrating disconnect between the prevalence of obesity and formal diagnosis. The National Health and Medical Research Council (NH&MRC) guidelines (2013) for management of obesity in adults in Primary Care are structured around a five step process (the *‘5As’*; Ask & Assess, Advise, Assist and Arrange) [2]. This article aims to identify the key challenges and successes associated with the *‘5As’* approach, to better understand the reasons for the gap between obesity and a diagnosis of obesity.

## Main text

### ‘Ask’

Obesity management guidelines are based upon the importance of conducting a brief intervention, to achieve a modest sustained and monitored weight reduction of 5–10% annually to bring about health benefits [2]. Ideally this will occur once a therapeutic relationship has been established, to ensure care provisions are ‘…person-centered, culturally sensitive, non-directive and non-judgmental’ [2]. General Practitioners’ (GPs) are the primary source of care for people with long term health conditions [3] and 87% of Australians are reported to access their GP at least once a year [4]. Poised in this vital position therefore, GPs are not only ideally placed, but entrusted by best practice to measure obesity predictors, diagnose obesity and monitor progress [2, 5]. Laidlaw et al. (2015) reported that although GPs initiated weight discussions more often than patients did, their attempts were often blocked by the patients [6].

‘Asking’ a patient about their weight may prove challenging for some Australian GPs, who report having difficulty accurately recognising aberrant body weight; 12% of obese people in one study were categorised as non-overweight by their GP [7]. This may be an artifact of a wider societal normalisation of obesity, a phenomenon which may also influence health professionals who are not immune to such perceptions. High rates of obesity are steering public perception, allowing an unhealthy weight to become socially conditioned as normal, thus altering the ability to identify risk [8].

### ‘Assess’

The NHMRC guidelines for the management of overweight and obesity in Primary Care suggest that clinicians measure waist circumference in addition to height and weight to calculate BMI as the best mode of assessing obesity in adults [2]. Assessing weight predictors however, appears to be poorly conducted according to a recent Victorian metropolitan study investigating the primary care medical records of over 270,000 adults which found that only 22.2% of people had a BMI recorded and only 4.3% had a waist circumference recorded [9]. GPs may be reluctant to measure weight and waist circumference, and calculate BMI due to insufficient time during consultations or for fear of offending patients [10]. Campbell et al. found that although GPs considered themselves well prepared to treat obesity, they considered weight management processes have limited efficacy and found it ‘professionally unrewarding’ [11]. In their exploration of the way obesity is currently managed in primary care, Sturgiss and colleagues concur that GPs should have the role of care coordinators in obesity management in line with current national guidelines, yet the reduced availability of allied health care, especially specialised obesity care can make this difficult, particularly in rural areas [12]. Gordon and Black agree that being equipped to have a conversation with patients about obesity is more effective if local services are available to assist with treatment strategies [13]. Are GPs therefore reluctant to assess if they feel that little can be done?

### ‘Advise’

‘Advising’ a patient to lose weight is worthwhile. When a doctor names and discusses obesity, patients are more likely to have a realistic perception of their weight and want to do something about it [14, 15]. Doctors themselves are not exempt from being overweight or obese which may prevent them from actively diagnosing their obese patients or, their patients taking their advice seriously [16, 17]. Interestingly however, one study showed that patients had greater trust in nutrition advice given by an overweight doctor than a doctor of normal BMI [18, 19]. GP communication style and the language used can impact adherence [20] and the likelihood of successful weight loss [21]. If a patient perceives that they are being judged about their weight, they are likely to attempt weight loss, but are less likely to achieve a clinically significant reduction in weight [19].

### ‘Assist’

‘Assisting’ patients to achieve their goals is hampered by barriers. While recent evidence has demonstrated that doctors are more likely to address the issue of weight gain with obese patients than overweight patients [22], it may be deemed more advantageous to intervene at an earlier stage. Patient engagement in weight loss is crucial [23], however some health professionals may harbor negative attitudes toward obese people and may view obese patients as non-adherent, lazy and having poor self-discipline [24]. Such weight related stigma or weight bias among health professionals can lead to failure to advise patients and treat obesity [25] a reality that cannot be ignored [26]. Australian GPs report a need for additional support in treating obesity, such as structured tools to assist in overcoming their lack of confidence in obesity management [27]. Furthermore, the same study discussed the prospect of greater self-efficacy and a more authentic GP-patient interaction by refocussing on healthy lifestyle goals rather than weight loss in and of itself [27].

### ‘Arrange’

Chronic conditions in Australia can be treated by GPs through the initiation of a General Practitioner Management Plan (GPMP), a holistic Federally funded and subsidised plan that allows an ability to recruit allied health, such as Exercise Physiology, Dietetics or Psychologists, to provide goal directed care as a team [28]. Meeting the criteria of this plan however, leaves much to interpretation, *“…a chronic medical condition is one that has been (or is likely to be) present for six months or longer, for example, asthma, cancer, heart disease, diabetes, arthritis and stroke*” [28]. Is obesity a risk factor or a chronic condition in its own right? The relevance of this question is pertinent to either active treatment with a formalised approach or hesitation. The pathogenicity of obesity is known to result from a complex and insidious interplay between behavioural, social, environmental and biological (genetic and epigenetic) factors that regulate energy balance and fat stores [29–31]. Therefore, it may be argued that obesity is indeed a chronic condition.

Accordingly, the World Health Organisation (WHO), the European, Canadian and American Medical Associations have acknowledged obesity as a chronic condition, that like other chronic illnesses requires a life-long commitment to a complex weight management regime [31, 32]. Obesity Australia (OA), a national Scientific Advisory Council advocating for a change in public perceptions about obesity is calling for the Australian Medical Association (AMA) to follow the directive of our international peers and define obesity as a chronic condition [33]. To date this has not occurred. Currently, the *‘Royal Australian College of General Practitioner Guidelines for preventive activities in general practice 8* ^*th*^ *Edition’* indicates that overweight and obesity are risk factors for chronic conditions [5], thus failing to meet the defining requirements of a GPMP. Unfortunately the recent Commonwealth inquiry into Chronic Disease Prevention and Management in Primary Health Care [34], fell short of resolving this issue. While a raft of modifiable risk factors for chronic conditions have been agreed upon, namely: tobacco smoking, alcohol misuse, physical inactivity and poor nutrition [34], obesity is barely mentioned. Limited only to a notation by the Dietitians Association of Australia adding that *‘the impact of resultant or associated obesity on these risk factors is significant as well, with the effects on a patient’s risk of chronic disease increasing with each risk factor present in their life’ p28* [34].

Pleasingly, a study conducted in New Zealand found that GPs were aware that it was their role to provide care for obese people [35]. Establishing such care routinely for all overweight and obese people is the challenge. Onsulana and colleagues (2015) conducted a randomized control trial in Canada evaluating the effectiveness of implementing the 5As in conjunction with weight management shared decision making (SDM) tools [36]. Clinicians identified that pictorial tools and fact sheets that offer the opportunity for primary health care teams to convey a rapid yet consistent message to patients, assisted in entrenching obesity management into routine care [36].

The Canadian Medical Association (CMA) has suggested that defining obesity as a chronic condition offers a formalised process for GPs that officially alters thinking, detaching obesity from the ‘lifestyle risk factor’ mindset to a belief that concedes a medicalised treatment obligation [31]. Medical hesitation, especially around assessment, consequently challenges public health attempts to mitigate social conditioning amid the current obesity ‘epidemic’ in Australia (27.5%), a proportion which has increased a staggering 47% since 1995 [33]. Clear guidelines are essential; however, their impact will be limited in addressing this problem alone, especially if Australian primary care clinicians feel inadequately equipped to manage obesity.

## Conclusion

This paper identifies multiple challenges in addressing the burden of obesity in Australia. Firstly, strong evidence suggests that declaring obesity as a chronic condition is vital in overcoming medical ambivalence and hesitation. Secondly, practitioners must feel confident and competent in treating obesity using the 5As approach and other SDM tools, which may require a medical commitment to providing professional development opportunities. Without this, it is difficult to foresee a shift in community attitudes toward both the stigma that surrounds obesity and the normalisation of an overweight and obese Australia.

## Abbreviations

BMI: Body Mass Index
NH&MRC: National Health and Medical Research Council
5As: Ask, Assess, Advise, Assist, Arrange
GPQ: General Practitioner
GPMP: General Practitioner Management Plan
WHO: World Health Organisation
OA: Obesity Australia
AMA: Australian Medical Association
SDM: Shared decision making
CMA: Canadian Medical Association
